# 

**Published:** 1892-01

**Authors:** 


### CONTENTS FOR JANUARY, 1892.

ORIGINAL COMMUNICATIONS.

The Prevention of Tuberculosis. By Henry R. Hopkins, M. D. .... 321
Ointments and Pastes. By Ernest Wende, M. D. .... 325

CLINICAL REPORTS.

A Case of Syphilodermata, Accompanied by Peculiar Symptoms .... 330

SOCIETY PROCEEDINGS.

Transactions of the Fourth Annual Meeting of the American Association of Obstetricians and Gynecologists .... 333

PROGRESS IN MEDICAL SCIENCE.

Physiological Chemistry .... 349
Ophthalmology .... 356
Medical Jurisprudence .... 358

NEW INSTRUMENTS.

A New and Improved Form of Club-Foot Shoe .... 361

EDITORIAL.

The Street Cars and the Public Health .... 362
Editorial Retirement of Dr. Mundé .... 363
Sanitary Condition of Public Schools .... 364
Ohio Moves for State License .... 365

OBITUARY.

Dr. Lucien Damainville—Dr. Henry Fraser Campbell .... 366
Dr. Simeon Tucker Clark—Dr. William Chace .... 383

PERSONAL.

Dr. Nelson Saunders—Dr. George Ben Johnson—Dr. W. A. Putnam .... 367
Dr. Daniel Lewis .... 383

MEDICAL COLLEGE NOTES.

Cincinnati College of Medicine and Surgery .... 368
University of Buffalo, Department of Pharmacy .... 369
Mrs. C. P. Huntington .... 369

MEDICAL SOCIETIES.

American Pharmaceutical Association .... 369
Session of the Association of Military Surgeons .... 370

Book Reviews .... 370
Books and Pamphlets Received .... 380
Miscellany .... 384

